# Efficacy is Not Everything: Eliciting Women’s Preferences for a Vaginal HIV Prevention Product Using a Discrete-Choice Experiment

**DOI:** 10.1007/s10461-019-02715-1

**Published:** 2019-11-06

**Authors:** Erica N. Browne, Elizabeth T. Montgomery, Carol Mansfield, Marco Boeri, Brennan Mange, Mags Beksinska, Jill L. Schwartz, Meredith R. Clark, Gustavo F. Doncel, Jenni Smit, Zvavahera M. Chirenje, Ariane van der Straten

**Affiliations:** 1 Women’s Global Health Imperative, RTI International, 351 California Street, Suite 500, San Francisco, CA 94104 USA; 2 Health Solutions, RTI International, Research Triangle Park, NC USA; 3 MatCH Research Unit (MRU), Faculty of Health Sciences, University of the Witwatersrand, Durban, South Africa; 4 CONRAD, Eastern Virginia Medical School, Arlington, VA USA; 5 University of Zimbabwe College of Health Sciences Clinical Trials Research Centre, Harare, Zimbabwe; 6 Department of Medicine, Center for AIDS Prevention Studies, University of California, San Francisco, CA USA

**Keywords:** HIV prevention, Discrete-choice experiment, South Africa, Zimbabwe, Women

## Abstract

As new female-initiated HIV prevention products enter development, it is crucial to incorporate women’s preferences to ensure products will be desired, accepted, and used. A discrete-choice experiment was designed to assess the relative importance of six attributes to stated choice of a vaginally delivered HIV prevention product. Sexually active women in South Africa and Zimbabwe aged 18–30 were recruited from two samples: product-experienced women from a randomized trial of four vaginal placebo forms and product-naïve community members. In a tablet-administered survey, 395 women chose between two hypothetical products over eight choice sets. Efficacy was the most important, but there were identifiable preferences among other attributes. Women preferred a product that also prevented pregnancy and caused some wetness (p < 0.001). They disliked a daily-use product (p = 0.002) and insertion by finger (p = 0.002). Although efficacy drove preference, wetness, pregnancy prevention, and dosing regimen were influential to stated choice of a product, and women were willing to trade some level of efficacy to have other more desired attributes.

**Electronic supplementary material:**

The online version of this article (10.1007/s10461-019-02715-1) contains supplementary material, which is available to authorized users.

## Introduction

In several recent trials of novel HIV prevention products targeting women, low adherence was found to be a critical issue impacting adequate protection against HIV [1–4.] Reasons for low adherence have been attributed to multiple factors, including the product’s mode and frequency of administration, associated adverse events, and partner influence [5, 6.] Information about women’s preferences for features of an HIV prevention product could help minimize issues of adherence and benefit product development. By preemptively designing new technologies that incorporate users’ preferences, the product may be more attractive and acceptable and used more consistently.

End-users’ preference assessments have become common practice in evaluating new treatments. In health economics, and increasingly in HIV prevention research, discrete-choice experiments (DCEs) are used to elicit preferences by asking participants to state their choice between alternative product designs [7–14.] DCEs are based on the assumption that goods and services can be described by a set of features or “attributes,” which have a number of variations or “levels,” and that the overall preference for a product can be determined by the sum of individual preferences for the product’s attributes. In a DCE, the relative importance of attributes is estimated by observing trade-off decisions between series of hypothetical products with varying attribute levels [15.]

The Quatro Study was a mixed-method study in South Africa and Zimbabwe designed to examine the acceptability of four vaginally delivered HIV prevention products in the form of a gel, insert, film, and ring [16.] A DCE was developed to further explore women’s preferences beyond the four product formulations studied. In this paper we explore the features of a vaginal product that were important to women using the DCE methodology to inform future HIV prevention product development.

## Methods

### Study Sample

The survey was conducted at two research clinics—in Durban, South Africa and in Chitungwiza, Zimbabwe—and included two distinct samples at each site: product-experienced and product-naïve women. The product-experienced sample were women recruited directly from the Quatro clinical study, a randomized crossover study of four vaginal placebo HIV prevention forms: a monthly ring and precoitally inserted gel, tablet-like insert, and film (NCT02602366). Details of the crossover study and sample recruitment can be found in Montgomery et al. [16] In short, women used each product for 1 month in a randomized sequence and chose their preferred product to use for an additional month. The product-naïve sample was recruited from the same communities, but they did not participate in the crossover study. Women aged 18–30, non-pregnant, HIV negative, and sexually active (defined as vaginal sex ≥ 3 times per month in past 3 months) and with no prior participation in microbicide or pre-exposure prophylaxis (PrEP) research were eligible to participate. All participants completed the survey between November 2016 and June 2017. Local ethics and regulatory committees in South Africa and Zimbabwe approved the survey, and all women provided written informed consent.

### Development of the DCE

A DCE survey includes a series of choice-set questions in which respondents are asked to choose between hypothetical product profiles. Each hypothetical profile is defined by a set of attributes and levels. Formative research was used to guide development of the DCE with 15 women aged 18–30 at each site. These participants were presented with 14 distinct attributes thought to be influential to choosing an HIV prevention product, based on literature review and the team’s previous research. Using a pile-sort technique, with each attribute listed on an individual card, women rated each attribute as “very,” “somewhat,” or “not” important by placing it into one of three piles. The five attributes ranked, on average, as most important were: effectiveness, how you use it, how often you have to use it, where you get it, and side effects. Privacy, partner preference, and color were on average rated least important. The ranking exercise contributed to attribute selection and highlighted how to refine explanations and imagery to ensure consistent conceptual understanding. Ultimately, the attributes included in the DCE were those thought to be modifiable from a product developer’s perspective.

Six attributes were selected for the DCE: dosing regimen (how frequently the product is inserted), mode of insertion (with or without applicator), vaginal wetness, partner’s awareness during sex, HIV protection, and pregnancy prevention. Table 1 summarizes each attribute level, including the description presented to participants with corresponding images. Formative research participants were excluded from participating in the DCE survey.

**Table 1:**
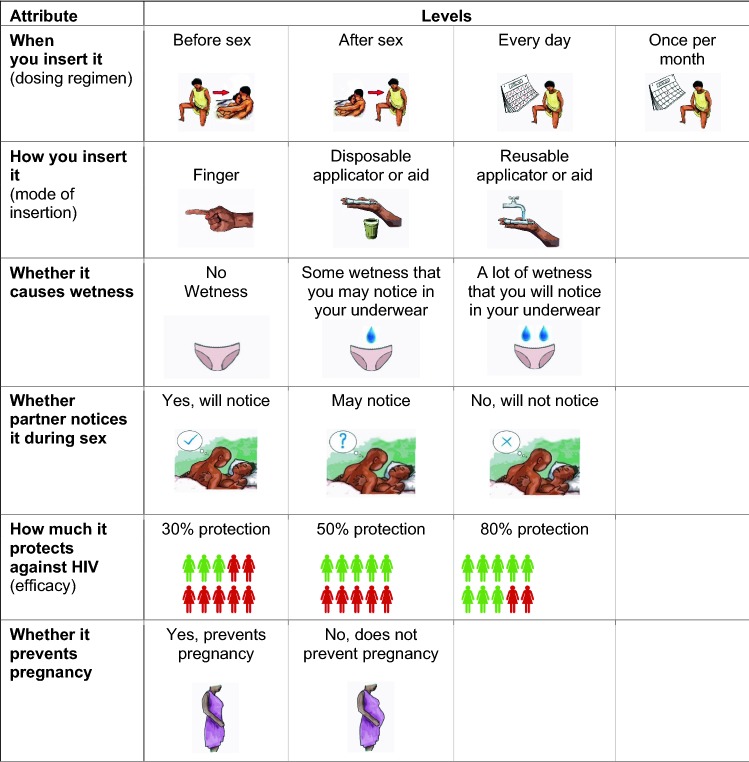
Discrete-choice experiment attributes, with corresponding images and text for each attribute level

### DCE Design and Survey Instrument

The combination of levels across attributes that respondents evaluate in a DCE is known as the experimental design. These combinations must have statistical properties to estimate preference weights of interest. The design was created following good research practices in SAS (software version 9.4; Cary, NC), using a D-efficient algorithm to construct a fractional factorial experimental design [17–19.] The full design consisted of 48 choice-set questions, which were divided into six blocks of eight questions. Each participant was randomized to one of the blocks and answered eight choice-set questions to limit respondent burden. In each question, participants were asked to choose between two hypothetical vaginally inserted HIV prevention products with varying combinations of attribute levels. Supplemental Fig. 1 illustrates an example choice-set.

The survey was administered on tablet computers and programmed in English, Zulu, and Shona using Open Data Kit software [20.] With assistance from the interviewer, the participant was first thoroughly introduced to each attribute individually, along with its corresponding levels. Each level had a unique image and text. After each attribute introduction, participants were asked a comprehension question regarding the attribute’s images and continued to the next attribute only once answered correctly. The participant was then asked one practice choice-set question to allow for any clarification of the task before independently completing the DCE. After the choice-sets, women were asked directly about their preferences through a series of multiple-choice questions. In addition, they responded to demographic, sexual history, and HIV risk perception questions.

### Sample Size and Analysis

Minimum sample size requirements for DCEs are difficult to estimate and depend on several criteria, such as the number of attribute levels, question format, and need to conduct subgroup analyses. Based on our design, 200 participants per subgroup was recommended, which is in line with previous studies [21.] Hence our target sample size was 400 participants to allow for subgroup analysis by product experience.

Preference weights for each of the attribute levels were estimated using a random-parameters logit (RPL) model. RPL models are regularly used to analyze preference data and account for respondent heterogeneity by estimating a distribution of the preference for each attribute level [22.] All attributes were included as effects-coded categorical variables. With effects-coding, the estimate for the omitted level is the negative sum of the included levels’ estimates, and zero represents the mean effect of all levels of the attribute (as opposed to the omitted level as in dummy coding) [23.] The RPL model calculates normalized mean preference weights, which represent the relative preference for each level in relation to the mean attribute effect. All levels were estimated as random parameters with a normal distribution. Because data were collected from several sources (i.e., from two countries, and within country by product experience), separate models were first estimated for each source, and a test by Swait and Louviere was used to confirm whether data from different sources could be analyzed together [24.] In addition, we conducted subgroup analyses to test for differences in preferences by education (completed secondary school), age (< 25 years), and frequency of sexual activity (> 8 vaginal sex acts in the past month). Each subgroup analysis was conducted by interacting every attribute level in the model with a dummy-coded variable identifying respondents of the subgroup and adding all interaction terms to the original RPL model. The estimated parameters on the interaction terms can be interpreted as the difference in preferences between the subgroup of interest and the reference group. A Wald test was used to test for joint significance of interaction terms. P-values were not adjusted for multiple comparisons as subgroup analyses were exploratory; p < 0.05 was considered significant.

Lastly, the preference weights from the main RPL model were used to predict the relative share of the sample that would have chosen between products with varying attribute levels. A hypothetical product with higher efficacy but less preferred other attribute levels was compared to a moderately effective product with more preferred other levels.

Responses to direct elicitation of preferences and sociodemographic questions were summarized by frequencies and percentages for categorical outcomes or means for continuous outcomes. Chi square tests or t-tests were used to compare responses between countries. All analyses were conducted using Stata 15.0 (StataCorp, College Station, Texas).

## Results

In total, 395 women completed the DCE—201 in Zimbabwe and 194 in South Africa. Overall 44% were product-experienced (i.e., had used all four vaginal placebo products in the Quatro clinical study). The remaining 56% were product-naïve. The median age was 24 years old (interquartile range 21-26 years). Nearly all participants had a primary partner (97%), and most had completed secondary school (69%). Women from the two research sites differed significantly on several characteristics evaluated (Table 2). More women in Zimbabwe were married or cohabitating (96% versus 9% in South Africa) and reported more vaginal sex acts in the past month (median 18 versus 4.5). Most women in South Africa had used a male condom (97%) and an injectable method (75%) for family planning, whereas in Zimbabwe, most had used oral pills (82%). Only six women had ever taken oral PrEP (five in South Africa, one in Zimbabwe). When asked about preferences for the vaginal environment during sex, more women in Zimbabwe preferred their vagina to be dry (58% versus 25%, p < 0.001).

**Table 2:** Characteristics of discrete-choice experiment participants, by country

	Durban, South Africa (N = 194)	Chitungwiza, Zimbabwe (N = 201)	Total (N = 395)
	N (%)	N (%)	N (%)
Sample population			
Product-experienced^a^	82 (42)	91 (45)	173 (44)
Product-naïve	112 (58)	110 (55)	222 (56)
Age, years			
Median (IQR)	23 (20–26)	25 (22–27)	24 (21–26)
Age 25–30 years**	65 (34)	111 (55)	176 (45)
Has primary partner*	184 (95)	199 (99)	383 (97)
Lives with partner or married**	18 (9)	193 (96)	211 (53)
Has casual sex partner**	29 (15)	3 (2)	32 (8)
Ever exchanged sex	5 (3)	2 (1)	7 (2)
Parity > 0**	135 (70)	200 (100)	335 (85)
Completed secondary school*	148 (76)	124 (62)	272 (69)
Earns an income**	34 (18)	94 (47)	128 (32)
No food insecurity past 4 weeks	100 (52)	116 (58)	216 (55)
Attend religious services at least once a week**	152 (78)	201 (100)	353 (89)
Family planning and/or HIV prevention methods ever used			
Male condom**	188 (97)	117 (58)	305 (77)
Oral pills**	50 (26)	165 (82)	215 (54)
Injectable**	146 (75)	67 (33)	213 (54)
Implants**	25 (13)	89 (44)	114 (29)
Female condom	15 (8)	17 (9)	32 (8)
Other vaginal product (gel/spermicide/diaphragm)	6 (3)	0 (0)	6 (2)
Number sex acts past month, median (IQR)**	4.5 (3–7)	18 (11–24)	8 (4–20)
Prefer vagina to be dry or wet during sex**			
Dry	48 (25)	116 (58)	164 (42)
Wet	146 (75)	85 (42)	231 (59)

*p < 0.05; **p < 0.001

^a^Participant of Quatro clinical study, a randomized crossover study of four vaginal placebo HIV prevention forms

### Stated Preferences

**Fig. 1:**
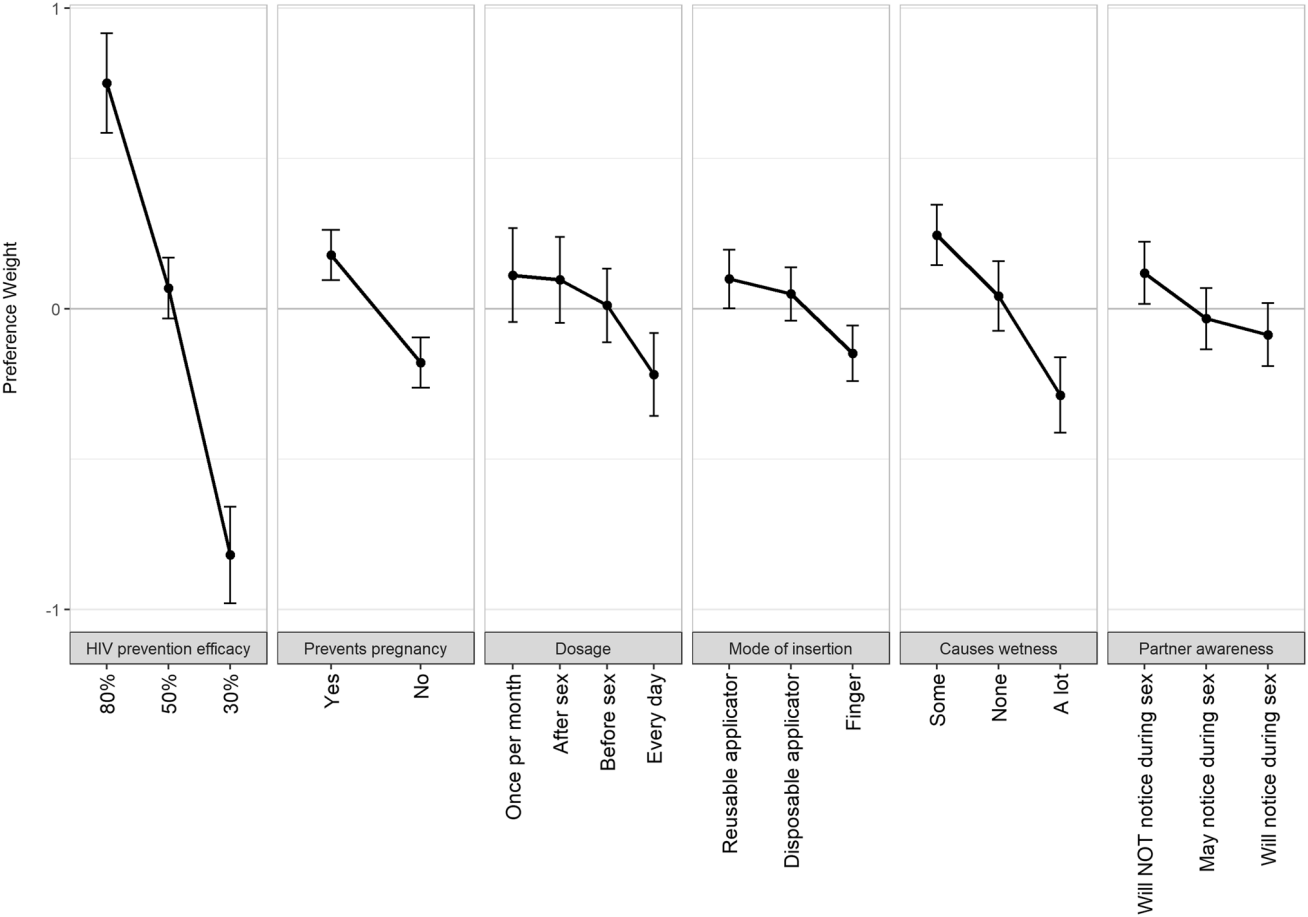
Normalized preference weights, with 95% confidence intervals, from the random-parameters logit model (N = 395)

The Swait and Louviere test confirmed that data from different sources could be analyzed together, since respondents from South Africa and Zimbabwe, as well as respondents with different product experience, did not present a significant difference in preferences (p > 0.90). Therefore, one RPL model was used to estimate preferences for all 395 participants. Normalized mean preference weights for each attribute level, with 95% confidence intervals, are depicted in Fig. 1. Large positive weight values indicate more preference and smaller negative values indicate less preference. The vertical distance between levels represents the relative strength of importance for each attribute. Table 3 provides the detailed results of the RPL model coefficients.

**Table 3:** Normalized random-parameters logit model coefficients, by attribute and level

	Coef.	SE	95% Confidence interval	p value^a^
HIV prevention efficacy				
30% protection	− 0.82	0.08	(− 0.98, − 0.66)	< 0.001
50% protection	0.07	0.05	(− 0.03, 0.17)	0.19
80% protection	0.75	0.08	(0.58, 0.92)	< 0.001
Prevents pregnancy				
Yes	0.18	0.04	(0.09, 0.26)	< 0.001
No	− 0.18	0.04	(− 0.26, − 0.09)	< 0.001
Dosing regimen				
Before sex	0.01	0.06	(− 0.11, 0.13)	0.86
After sex	0.10	0.07	(− 0.05, 0.24)	0.19
Every day	− 0.22	0.07	(− 0.36, − 0.08)	0.002
Once per month	0.11	0.08	(− 0.04, 0.27)	0.16
Mode of insertion				
Finger	− 0.15	0.05	(− 0.24, − 0.06)	0.002
Disposable applicator or aid	0.05	0.05	(− 0.04, 0.14)	0.29
Reusable applicator or aid	0.10	0.05	(0.00, 0.20)	0.05
Causes wetness				
No wetness	0.04	0.06	(− 0.07, 0.16)	0.47
Some wetness	0.24	0.05	(0.14, 0.34)	< 0.001
A lot of wetness	− 0.29	0.06	(− 0.40, − 0.16)	< 0.001
Partner awareness during sex				
Yes, will notice	− 0.09	0.05	(− 0.19, 0.02)	0.11
May notice	− 0.03	0.05	(− 0.14, 0.07)	0.53
No, will not notice	0.12	0.05	(0.02, 0.22)	0.02

*Coef* coefficient (normalized preference weight), *SE* standard error

^a^For test if attribute level preference is significantly different from average attribute effect (i.e. different from zero)

Among the six attributes used to characterize the vaginal HIV prevention products, women placed the most weight on efficacy when deciding between two products, with a strong preference for a product that provides 80% protection over 30% (p < 0.001). The other attributes had similar relative importance, with each having significant preferences among levels. On average, women disliked a product inserted every day (p = 0.002). There was slightly more preference for insertion after sex or once monthly compared to insertion before sex, although this difference was not statistically significant (p = 0.42). Women disliked a product inserted with a finger compared to with an applicator (p = 0.002). There was more preference for a product that causes some wetness over a product that causes a lot of wetness (p < 0.001). There was more preference for a product that will not be noticeable to a partner during sex (p = 0.02). Women also preferred a product that prevents pregnancy (p < 0.001). Therefore, all attributes influenced choice. Only 44 women (11%) chose the product with highest HIV protection in every choice set.

In addition to our initial evaluation by country and previous product experience, we assessed if preferences differed by age group, education level, and frequency of vaginal sex. There were no significant differences in preferences by completion of secondary school (p ≥ 0.05). Younger women (< 25 years) had stronger preferences related to mode of insertion, with less preference for finger insertion than older women (p = 0.04). Women who had more vaginal sex in the past month (> 8 acts) had significantly less preference for a product that needed to be inserted before sex (p = 0.005). Women who had less sex (≤ 8 acts) had greater dislike for a product inserted every day (p = 0.03).

### Preference Shares

**Fig. 2:**
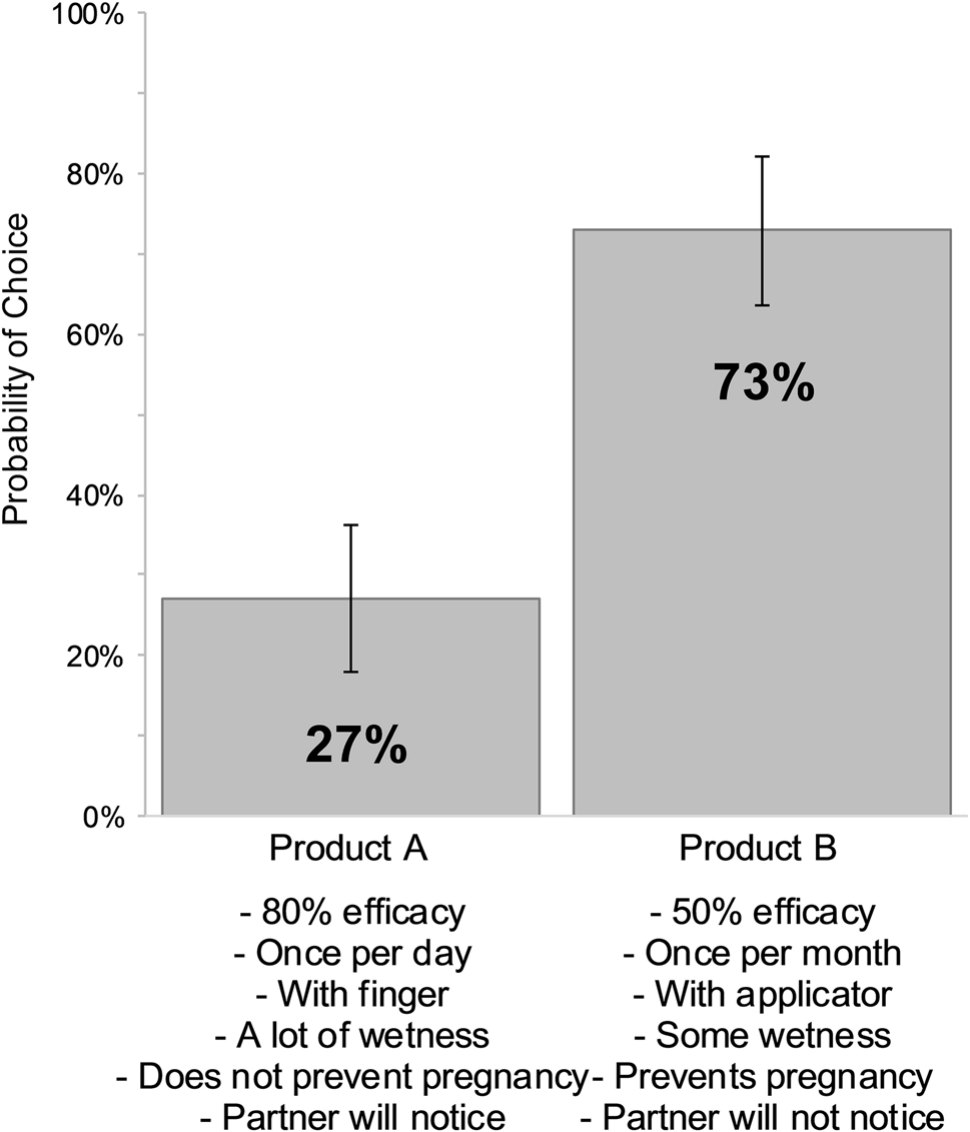
The estimated share who would have chosen between two hypothetical HIV prevention products using results from the random-parameters logit model. Product A: 80% protection, inserted once per day, using her finger, creates a lot of wetness that will be noticeable in her underwear, partner will notice during sex, and does not prevent pregnancy. Product B: 50% protection, inserted once per month, using a reusable applicator, creates some wetness that may be noticeable in her underwear, partner will not notice during sex, and also prevents pregnancy

Figure 2 presents preference shares for two hypothetical product profiles, calculated from the weights generated from the main RPL model. Preference shares help to evaluate collective preferences for combinations of attribute levels. To understand the influence of efficacy relative to other attributes, we constructed two product profiles: Product A with a high efficacy level and the least preferred levels of other attributes (i.e., inserted every day, finger insertion, causes a lot of wetness, does not prevent pregnancy, and partner will notice during sex), compared to Product B with moderate HIV protection (50%) but more preferred levels of other attributes.

In this scenario, the preference share was greater (73%, 95% CI 64%, 82%) for a product with only moderate efficacy but other favorable attributes, like inserted monthly, caused some wetness, prevented pregnancy, was unnoticeable to partner, and had a reusable applicator.

### Direct Elicitation of Preferences

After the DCE portion of the survey, participants were explicitly asked about their preferences for an HIV prevention product. They were asked to rank the following eight attributes in order from most to least important when choosing an HIV prevention product: cost, efficacy, how it is inserted, when it is inserted, where to get it, wetness, pregnancy prevention, and partner awareness. Two-thirds (67%) ranked how well it protects against HIV as the most important. The most common features ranked least important were partner awareness during sex (25%), whether it causes wetness (19%), and where to get it (16%). The importance of cost was distributed evenly, with equal proportions (17%) finding it most and least important.

Two-thirds of women in both countries (66%) said they would tell their primary partner they were using an HIV prevention product even if it could be used without him knowing. Most (86%) said they would rather use a 2-in-1 product that combined HIV and pregnancy prevention. However, 42% of South African and Zimbabwean women said they would be “extremely” or “very” unlikely to use a dual-purpose product if it caused irregular bleeding or spotting. Women affirmed they would want a product that made their vagina feel “warm” (95%), “tight” (93%), and “clean” (99%).

## Discussion

In a DCE survey designed to explore young women’s preferences for features of a vaginally delivered HIV prevention products in Southern Africa, efficacy was the most important feature driving stated choice of a product. However, all attributes assessed were important and considered when deciding between products. Preferences were framed more so by dislike for a particular attribute level. The dislike for certain features, such as daily insertion and a lot of wetness, was such that when combined in the same product, the sum of their negative weights outweighed the benefit of higher efficacy; meaning, the majority of women were willing to trade some level of efficacy to gain more desirable other attributes. This was a product-agnostic DCE in that we evaluated a series of attributes that could apply to a range of vaginally inserted product formulations. We did not find any influence of previous product experience on preferences, indicating that experience with the four vaginal products from the Quatro clinical study did not influence opinions of the product attributes assessed in this DCE. We also found that preferences were similar across countries.

Efficacy has been found to be most influential to choice of HIV prevention methods in previous DCE studies in the region [10, 12, 14.] HIV acquisition in sub-Saharan Africa continues to be high, particularly for young women [25,] and thus it is perhaps unsurprising that efficacy would be of paramount importance in this endemic context. The importance of efficacy could have implications for clinical trials, where lack of evidence regarding a new active product’s level of efficacy may impact participants’ motivation to use it. The levels of protection we explored were 80%, 50%, and 30%. The HIV prevention methods that are currently approved and available are condoms and oral PrEP, both of which have high effectiveness (> 80%) if correctly and consistently used [26–28.] Presuming that any product would need to have evidence of high efficacy for approval, the features of a product that influence its ability to be successfully integrated into the sexual and reproductive life of women are critical.

The ability of a product to protect from HIV does not mean that it will be widely accepted or used. For example, female condoms are efficacious, but some women have reported low acceptability because of difficulty with insertion and integration into sex, and they are non-discreet [15, 29.] The results of this DCE show that other attributes of a product, specifically dosing regimen, wetness, and pregnancy prevention, inform preference. Qualitative studies in Southern Africa found that women would consider HIV prevention options that align with their current sexual and reproductive health routines [30.] One aspect of this routine includes preferences and practices during sex [31.] In our sample, about 40% of young women, mostly in Zimbabwe, said they preferred their vagina be dry during sex. Not surprisingly then, we found that choice of an HIV prevention product was significantly influenced by the amount of wetness caused by the product. The importance of vaginal wetness has been previously reported in trials of vaginal gels, where leakage before/during/after sex was the most commonly reported issue with the formulation [32.] Furthermore, concerns with hygiene or discomfort with touching the vagina may explain the disinterest we found for a product inserted by finger, especially among younger women. Insertion applicators may be a more modifiable aspect of a product than changing dosing platform features [33.]

Pregnancy prevention, which has been shown in previous DCEs to be an important feature of HIV prevention methods [10, 12, 14,] was also a key consideration for product choice. Nearly all women said they would prefer a multipurpose prevention technology (MPT) that offers both HIV and pregnancy prevention, and some women were willing to choose a less efficacious product in order to have an MPT. Of note, several women indicated that the desirability for an MPT might change if it creates significant changes to the menstrual cycle. This feedback may need to be considered in the development of hormonal-based MPTs. Nevertheless, these data support the importance of continued research and development for MPTs.

Relative to other attributes presented in this DCE, partner awareness during sex was less influential to stated choice. This finding is seemingly inconsistent with qualitative findings from other studies that highlight the importance of male partners on women’s acceptance of HIV prevention products [30, 34.] For example, in a trial of the dapivirine vaginal ring, some women reported their low adherence was attributable to fears that their partners would oppose ring use or feel it during sex [35.] It may be that when asked to make a choice between products in isolation from male partners, women’s preferences are not influenced by her partner’s awareness; rather it is her use of the product that is impacted by her partner’s reactions. Alternatively, two-thirds of women said they would tell their partner about using an HIV prevention product even if it could be used discreetly; therefore, his awareness may be considered negligible when deciding between hypothetical products. It could also be that the wetness feature influences male perception of product use during sex, and preferences surrounding wetness confounds importance of partner awareness. A DCE with couples is underway that may illuminate how women’s preferences shift when male partner preferences and physical presence are incorporated [36.]

Finally, in this study we found no differences in preferences between women who had used vaginally inserted products for 5 months and women who were “product-naïve.” Other studies have similarly found no association between prior experience with a formulation and product preferences, including another DCE in South Africa [10, 37.] The product attributes included in this DCE were not indicative of one particular formulation that was actually used in the Quatro clinical study. Characterizing the Quatro study’s insert and film products with the attributes of this DCE would lead to nearly identical profiles; however, in the clinical study we found significantly different preferences by country for these two products (more women in Zimbabwe chose the film) [16.] Hence, we cannot extrapolate from the preferred attributes in the DCE what product formulation would have been chosen. Furthermore, product rankings in Quatro changed significantly on the individual level after women tried them, suggesting that actual use was indeed important to preferences [16.] DCE models characterize population stated preference, which may explain why individual-level preference changes are not noticeable. These population estimates can be valuable to product developers who are trying to make decisions on the best design for a larger market.

There are some limitations of this study. Women were recruited through convenience sampling, thus our findings might not be representative of all women in these communities. We did not include an opt-out option in the DCE, which would have allowed women to choose neither of the two hypothetical products presented. Therefore, we were not able to assess women’s interest in, or predicted uptake of, a vaginally delivered HIV prevention product. Because we did not include product form as an attribute, we were not able to assess preferences related to delivery platform. However, this design decision was purposeful so that we could examine features of products that could be adaptable to any formulation.

In this diverse sample of women in South Africa and Zimbabwe, both with and without experience using vaginal products, we identified preferences for features of a vaginally delivered HIV prevention product that were similar across samples. The results of this DCE add to the growing body of evidence that HIV prevention technologies must not only be effective but amenable to existing sexual and vaginal practices for a product to be desirable, chosen, and ultimately used.

## Electronic supplementary material

Below is the link to the electronic supplementary material.

Supplementary material 1 (TIFF 632 kb). **Supplemental Figure** **1**. An example of a choice-set question answered by a participant using a tablet device. Each participant answered eight choice-set questions
