# Supplementary figures and images for: Efficacy is Not Everything: Eliciting Women’s Preferences for a Vaginal HIV Prevention Product Using a Discrete-Choice Experiment
Source: AIDS Behav. 2019 Nov 6;24(5):1443–51. doi: 10.1007/s10461-019-02715-1 (PMC6990865; doi:10.1007/s10461-019-02715-1)

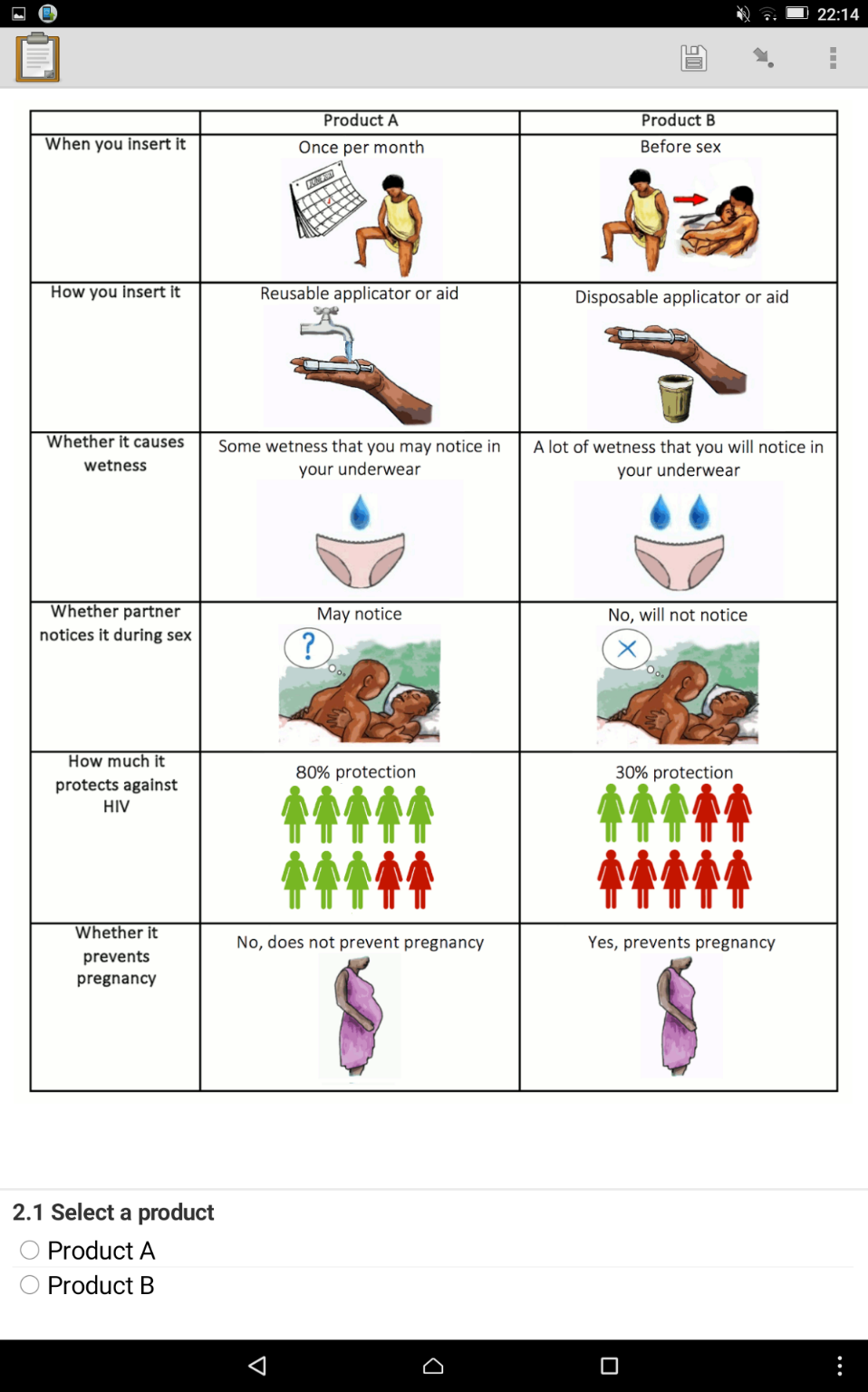

Supplement: Supplementary file 1 — Supplementary material 1 (TIFF 632 kb). Supplemental Figure 1. An example of a choice-set question answered by a participant using a tablet device. Each participant answered eight choice-set questions [file 10461_2019_2715_MOESM1_ESM.tif]
